# Psilocybin reduces low frequency oscillatory power and neuronal phase-locking in the anterior cingulate cortex of awake rodents

**DOI:** 10.1038/s41598-022-16325-w

**Published:** 2022-07-26

**Authors:** Caroline T. Golden, Paul Chadderton

**Affiliations:** 1 Department of Bioengineering and Centre for Neurotechnology, Imperial College London, London, SW7 2AZ UK; 2 School of Physiology Pharmacology and Neuroscience, University of Bristol, Bristol, BS8 1TD UK

**Keywords:** Neural circuits, Neuronal physiology, Depression, Post-traumatic stress disorder

## Abstract

Psilocybin is a hallucinogenic compound that is showing promise in the ability to treat neurological conditions such as depression and post-traumatic stress disorder. There have been several investigations into the neural correlates of psilocybin administration using non-invasive methods, however, there has yet to be an invasive study of the mechanism of action in awake rodents. Using multi-unit extracellular recordings, we recorded local field potential and spiking activity from populations of neurons in the anterior cingulate cortex of awake mice during the administration of psilocybin (2 mg/kg). The power of low frequency bands in the local field potential was found to significantly decrease in response to psilocybin administration, whilst gamma band activity trended towards an increase. The population firing rate was found to increase overall, with just under half of individual neurons showing a significant increase. Psilocybin significantly decreased the level of phase modulation of cells with each neural frequency band except high-gamma oscillations, consistent with a desynchronization of cortical populations. Furthermore, bursting behavior was altered in a subset of cells, with both positive and negative changes in the rate of bursting. Neurons that increased their burst firing following psilocybin administration were highly likely to transition from a phase-modulated to a phase unmodulated state. Taken together, psilocybin reduces low frequency oscillatory power, increases overall firing rates and desynchronizes local neural activity. These findings are consistent with dissolution of the default mode network under psilocybin, and may be indicative of disruption of top-down processing in the acute psychedelic state.

## Introduction

Psilocybin (4-phosphoryloxy-*N*, *N*-dimethyltryptamine) is a hallucinogenic compound, found in “magic mushrooms”, that can cause profound alterations in sensory perception, mood, and cognition^1^. Recently, there has been evidence to show that psilocybin may have a therapeutic benefit in treatmentresistant depression^2,3^, yet much is to be discovered about the mechanism of action of such effects and the altered state of consciousness that psilocybin produces.

Psilocybin is the pro-drug of psilocin, an indolealkylamine (IAA) that belongs to a group of hallucinogenic tryptamines^4^. These compounds act universally on the serotonergic (5-HT) system^5^, with a particular affinity for 5-HT_2A_ receptors^6^. Psilocybin primarily acts on 5-HT_2A_ and 5-HT_1A_ receptors^4^, both of which are expressed in the prefrontal cortex (PFC)^7^**,** however 5-HT_2A_ receptors are the only receptors with causal evidence for psychotomimetic effects^8^. Activation of 5-HT_1A_ receptors mediates membrane potential hyperpolarization^9,10^, whilst 5-HT_2A_ receptors have depolarising actions^9^. Approximately 61% and 51% of pyramidal PFC neurons express 5-HT_1A_ and 5-HT_2A_ receptors respectively^7^, with 80% exhibiting co-expression^11^. Parvalbumin-positive (PV+) interneurons have distinct populations that express 5-HT_1A_ and 5-HT_2A_ receptors^12^. In cortical layer V, ~ 30% of interneurons express one of the two types of receptors^13^. The mixed distribution and opposing actions of these two receptor subtypes make it difficult to infer the network action of psilocybin within the PFC. Accordingly, the physiological effects of psilocybin are unclear, with different imaging modalities providing divergent results. An early PET study found evidence of increased cortical metabolism in the PFC following psilocybin administration^14^, whilst more recent fMRI studies in both humans and rats have found BOLD signal reductions in cingulate cortex^15,16^, with the anterior cingulate cortex (ACC) demonstrating some of the largest reductions.

There has been evidence for a change in functional connectivity between cortical brain regions in response to psilocybin. In fMRI studies, psilocybin appears to alter global functional connectivity patterns by increasing connectivity between regions of the PFC associated with the Default Mode Network (DMN) and regions related to the Task Positive Network (TPN) during resting state after injection^17^, whilst decreasing connectivity between the PFC and the posterior cingulate cortex^15^. Psilocybin has also been shown to decrease connectivity levels in the DMN^15,16,18^. It has been hypothesized that such changes in connectivity may be linked to altered states of consciousness^15^, and are a prediction of the theory of the entropic brain under psilocybin as proposed by Carhart-Harris et al.^19^. However, the underlying mechanisms and the method by which psilocybin affects cortical circuits on a neuronal level are unknown.

The ACC plays a key role in processing negative emotions such as fear and anxiety, particularly the appraisal of fear-evoking or anxiety-evoking stimuli, as evidenced by both human and animal studies (see Etkin et al., 2011^20^ for an in-depth review). Moreover, in humans, BOLD signals in the dorsal ACC have been shown to correlate with a shift in attention to interoceptive perception^21^. It is worth noting that the ACC has functional connectivity with important emotional processing areas within the brain, such as the amygdala and the periaqueductal grey^22,23^. Taken together, with the move towards using psilocybin as a therapeutic compound for conditions such as depression and post-traumatic stress disorder (PTSD), it is important to understand more about the impact that it has on the ACC.

### Investigating circuit action of psilocybin in ACC

LFP and multiunit activity (MUA) provide insights into changes in synaptic and spiking activity within local networks. Psilocybin administration has been associated with reductions in LFP power in low frequency bands, namely delta, theta, and alpha^18,24^. On a neural population level, the only evidence currently known derives from a study whereby psilocin was applied locally to the raphe nuclei via microiontophoresis, resulting in an inhibition of serotonergic neurons^25^. Whilst informative, the potentially differing distribution of 5-HT_2A_ receptors amongst pyramidal and interneuron populations of the ACC may result in quite different modulation profiles.

The field of psychedelic research has expanded in recent years, particularly in the region of therapeutic benefit. This necessitates a greater need for understanding the mechanism of action of compounds such as psilocybin. Electrophysiology can provide neuron and network-specific information local to the region of interest. Therefore, to characterize the effect of psilocybin at a dose of 2 mg/kg on the ACC, a set of experiments were performed on awake, head-fixed mice on a treadmill setup. The dose was chosen based on evidence from previous literature that psilocin dosed at 2 mg/kg induced a neurophysiological change in rats^16^. Our findings show significant reductions in the power of low frequency neural oscillations with concomitant trending increase in gamma oscillatory power. The activity of the network is increased under psilocybin, with a broad loss of synchronization between spiking activity and local field potential that is linked to a change in burst firing activity.

## Materials and methods

### Animals

Experiments were conducted on male and female C57 BL6 mice (n = 10, 5 female; Charles River UK Ltd & Harlan UK Ltd), and homozygous PValb-IRES-Cre mice (JAX), aged 6–14 weeks, weighing 18–25 g. Animals were housed under a 12/12 h light–dark cycle with food and water available ad libitum. Habituation and recordings were performed during the dark phase of the cycle. We selected a small sample size because effects of psilocybin on cortical population activity have been rarely evaluated in vivo. Thus, the initial intention was to gather basic evidence regarding psilocybin’s influence on neuronal firing and local field potential. All experiments were conducted under the UK Animals (Scientific Procedures) Act 1986. The study is reported in accordance with the ARRIVE guidelines.

### Surgical procedures

Mice were anaesthetised for all surgical procedures. Mice were placed in an induction chamber, into which 5% v/v isoflurane (Harvard Apparatus Ltd) was administered via inhalation using oxygen (1.2–1.6 L/min). Once anaesthetized the concentration of isoflurane was lowered to 1–2% v/v to maintain anaesthesia for the duration of the surgery. The body temperature of the mouse was maintained at 37 ± 0.5 °C using a homeothermic heat mat (DC Temperature Control System, FHC). Corneal drying was prevented using an ocular lubricant (Lacrilube, Allergen, UK). A custom-made plastic head-implant was fixed to the exposed cranium using tissue glue (Histoacryl, Braun Corporation, USA) and dental cement (Associate Dental Products Ltd). The hollow centre of the implant was positioned at the location of the future craniotomy ([1 2] mm AP, [−1 1] mm ML). Ground and reference pins were inserted above the cerebellum, and secured using dental cement. The exposed skull was then covered with Kwikcast (World Precision Instruments). Analgesia was provided via intraperitoneal (i. p.) injection of carprofen (5 mg/kg) into the shoulder of the mouse, and antibiotics (Baytril, dose: 250μL of 1 mg/ml solution, 6 mg enrofloxacin) were administered into the alternate shoulder, approximately 10 min before the removal of the anaesthetic, followed by the administration of buprenorphine jelly (Vetergesic containing 0.3 mg/ml buprenorphine in Hartley’s Jelly, dose: 0.8 mg/kg every 8–12 h as required) orally for 2–5 days post-operation. Following general anaesthesia, mice were placed in a recovery heat chamber for approx. 1 h, with food and water provided ad libitum. Mice were then returned to their home cages and monitored closely for the subsequent 24 h.

### Experimental setup and electrophysiology

The experimental setup was composed of a treadmill with a headplate fixation mount. Mice were initially habituated to the setup for 2–3 days prior to recordings to ensure they were comfortable on the treadmill. All hardware relating to the behaviour of the mouse was controlled using an Arduino Uno (Arduino). Electrophysiological recordings were performed using a ME64-FAI-MPA data acquisition system (Multichannel Systems, USA). Neuronal signals were sampled at 27 kHz and high-pass filtered at 0.5 Hz.

One hour prior to recording, mice were anaesthetized with isoflurane and a craniotomy was performed bilaterally (or occasionally unilaterally) across hemispheres. Electrophysiological recordings were performed on awake head-fixed mice that could sit comfortably in the setup. The probe (NeuroNexus A32 4 × 2 tetrode; shank spacing 150 μm, site area 200 μm^2^) was inserted in the coronal plane using the coordinates: AP 1.6 mm, ML: 0.5 mm, D: [0.8 1.1] mm^26^.

Baseline activity was defined as a 10 min period of spontaneous activity in the ACC prior to drug injection. Baseline recording began at a minimum of 25 min following probe insertion to allow the preparation to stabilize. Mice were briefly sedated using isoflurane during the injection period. The injection/anaesthetic period duration was 2–5 min (including the time for anesthesia recovery). A dose of 2 mg/kg was delivered via an intraperitoneal (i.p.) injection (n = 5). Control experiments were performed by injecting an equal volume of sterile saline (vehicle) and examining the pre- and post-injection effects (n = 5). The experimenter was blinded to the composition of the injection solution (i.e., psilocybin or vehicle). The time in which the effect of the vehicle or psilocybin was analyzed, referred to as the vehicle/psilocybin period was taken as a 10 min period, 20 min after the end of the injection period, to allow time for the compound to take effect. The locomotion of the mice was examined to ascertain whether the increase in population activity found in this paper was correlated with a change in speed. All mice showed little movement in both the baseline and psilocybin periods, hence there was no change between time spent running in between the two conditions (Wilcoxon signed-rank test: baseline vs. psilocybin, p = 0.44). One experiment was performed per animal, and each animal was sacrificed following experimentation. Recordings were made at similar times of day, and litter/cage mates were used where possible, in order to minimise the possibility of confounding differences between vehicle and psilocybin groups.

### Preparation of the systemic psilocybin solution and psychedelic dose verification

Psilocybin was dissolved in sterile saline using a sonicator for 1–2 h, to produce a solution of 0.4 mg/ml. Due to light-sensitivity, the solution was stored in darkened aliquots at −20 °C. This dose was verified to induce a head twitch response through observation in the home cage post-dosing. This was performed separately to the experiments presented here.

### Data analysis

All data was processed in Matlab (Mathworks). LFP was band-pass filtered to isolate individual frequency bands (BWdelta = 1–4 Hz, BWtheta = 4–8 Hz, BWalpha = 8–13 Hz, BWbeta = 13–30 Hz, BWlow-gamma = 35–55 Hz, BWhigh-gamma = 65–120 Hz), and subsequently down-sampled from 25 kHz to 500 Hz. The thresholds for low and high gamma were taken from Yamamoto et al. 2014^27^. Power spectral density was computed across 10 min of data (2 s sliding window, overlap = 0.9, nfft = 2^12^). The effect size (d) for t-tests was computed as Cohen’s d using a custom script (computeCohen_d(× 1, × 2,varargin); https://www.mathworks.com/matlabcentral/fileexchange/62957-computecohen_d-x1-x2-varargin; MATLAB Central File Exchange). The effect size (r) for the Wilcoxon signed rank test was calculated as r = Z/$\surd N$^28^. For population synchrony analysis, the method of Jones et al., was employed^29^. In brief, the local maxima and minima of the band-pass filtered data were detected to assign the peaks and troughs of the oscillation. Phase was linearly interpolated from trough (0°), to peak (180°), to trough (360°). The level of phase modulation within each frequency band was quantified using the concentration of the von Mises distribution (κ) for each cell^29,30^. The closer the concentration is to 0, the more it is reflective of a uniform distribution i.e. a phase unmodulated cell, the higher the concentration, the more it approaches a normal distribution and the more phase modulated the cell is. It was computed by modelling the distribution of spiking activity in each phase of a given neural frequency band as a Von Mises distribution. This distribution is characterized by μ, the center of the distribution i.e. the phase to which the neuron is tuned, and 1/κ, the variance. Hence, the reciprocal, κ, is a measure of the concentration of phase tuning (κ was calculated and the Rayleigh test was performed using custom scripts from CircStat^31^). Single units were isolated offline using the semi-automatic clustering software Klustasuite^32^. Of note, spike sorting was performed on the full experiment recording (> 1 h in each case). Clusters were rejected if they contained > 1% spikes within a 1 ms inter-spike interval^33^. Only single unit data were used in analysis. Data from one experiment that recorded 3 neurons was included in the LFP analysis but excluded from the single unit activity analysis to improve statistical representation. The baseline firing rate for z-score calculations was taken as the mean firing rate of a cell over all spatial bins (1 cm). The cumulative cell population from all psilocybin experiments (135 cells, N = 4 experiments) were analyzed to find those that exhibited burst firing. The method used to isolate bursting from single spikes was based on the approach developed by Ranck^34^ and used by Harris et al.^35^.

## Results

### The effects of psilocybin on LFP

**Figure 1:**
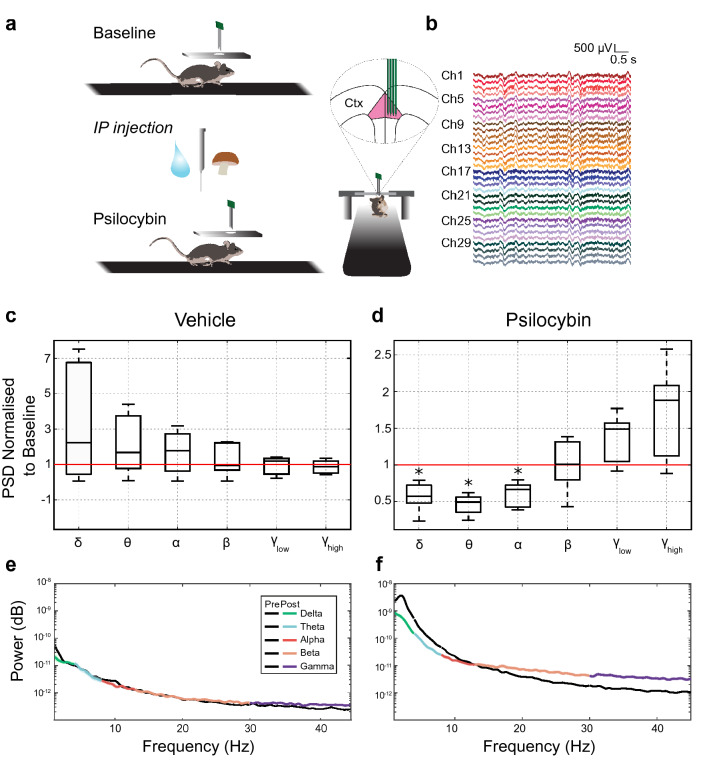
Redistribution of LFP power in ACC from low to high frequency bands following psilocybin administration. (**a**) Schematic of the experimental setup for recording from cerebral cortex (Ctx) before/after systemic administration of psilocybin in awake mice. (**b**) Example raw data recorded from ACC using 4 × 2 tetrode probe. Each trace represents a probe channel. Channels are colour-coded to highlight grouping by tetrodes. (**c**) Change in power spectral density (PSD) normalized to baseline for vehicle experiments for each neural frequency band (n = 5) (delta: *t*(4) = −0.98, *p* = 0.1967; theta: *t*(4) = −1.43, *p* = 0.2269; alpha: *t*(4) = −1.20, *p* = 0.2951; beta: *t*(4) = −0.65, *p* = 0.5493; low-gamma: *t*(4) = 0.29, *p* = 0.7893; high-gamma: *t*(4) = 0.77, *p* = 0.4827, paired sample t-test). (**d**) As in (**c**), for psilocybin experiments (delta: *t*(4) = 4.58, *p* = 0.0102, d = 2.86; theta: *t*(4) = 8.14, *p* = 0.0012, d = 5.25; alpha: *t*(4) = 5.15, *p* = 0.0067, d = 3.20; beta: *t*(4) = −0.04, *p* = 0.9717, d = −0.02; low-gamma: *t*(4) = −2.31, *p* = 0.0822, d = −1.46; high-gamma: *t*(4) = −2.31, *p* = 0.0802, d = −1.47, paired sample t-test). Asterisk denotes significance comparing the PSD in each frequency band between the pre- and post-conditions. (**e,f**) LFP power pre- and post-injection for vehicle and psilocybin experiments respectively. The baseline power for all frequency bands is in black.

To investigate the effects of psilocybin on network activity in the ACC, LFP was recorded to determine psilocybin-induced changes in each neural frequency band in awake, head-fixed mice on a novel treadmill setup (Fig. 1a,b). A baseline period of neuronal data was recorded from the ACC, followed by a 200 μl intraperitoneal psilocybin or vehicle injection (see “Materials and methods”). Neural frequency bands were isolated from raw LFP using band-pass filtering (delta: 1–4 Hz, theta: 4–8 Hz, alpha: 8–13 Hz, beta: 13–30 Hz, low gamma: 35–55 Hz, high gamma: 65–120 Hz), and power spectral density was computed in baseline and post-injection conditions for vehicle (n = 5) and psilocybin experiments (n = 5). Overall, psilocybin tilted spectral power away from low towards higher frequencies (Fig. 1d), with large effect sizes seen in delta (d = 2.86), theta (d = 5.25), alpha (d = 3.2) frequency bands. In accordance with previous findings in humans^18,24^, low frequency bands showed a significant decrease in power (delta: *t*(4) = 4.58, *p* = 0.0102, d = 2.86; theta: *t*(4) = 8.14, *p* = 0.0012, d = 5.25; alpha: *t*(4) = 5.15, *p* = 0.0067, d = 3.20, paired sample t-test), whilst the beta band remained unaffected (beta: *t*(4) = −0.04, *p* = 0.9717, d = −0.02). Power in low- and high-gamma bands trended towards an increase (low-gamma: *t*(4) = −2.31, *p* = 0.0822, d = −1.46; high-gamma: *t*(4) = −2.31, *p* = 0.0802, d = −1.47, paired sample t-test). No significant changes in spectral power were observed following injection of the vehicle (Fig. 1c,e). Thus, psilocybin administration is associated with a reduction of spectra power in the delta, theta and alpha range, and the transition to higher gamma power in the cortex (Fig. 1f). This effect mirrors the transition into a desynchronised state in awake, active animals whereby low frequency fluctuations decrease, and gamma power increases^36^.

### Psilocybin increases spiking and alters neuronal ensemble activity in ACC

**Figure 2:**
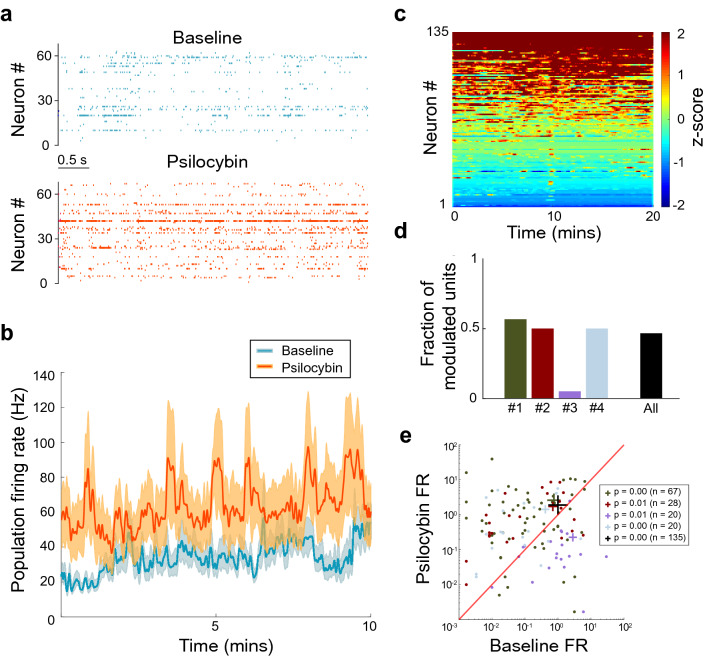
Psilocybin increases firing rates in ACC neurons. (**a**) Raster plots of neurons from a single experiment in the baseline and psilocybin condition. (**b**) Psilocybin increases firing rates in ACC populations from 34 ± 8 Hz in the baseline condition to 64 ± 19 Hz after drug administration (n = 135). The mean population firing rate represents the mean of the total spikes across neural populations. (**c**) Z-scored firing rate for each cell after psilocybin administration. Cells sorted according to peak score. Time 0 indicates the beginning of the “psilocybin period” as denoted in the “Materials and methods” section. (**d**) Proportion of cells significantly activated by psilocybin (3 + consecutive bins with z score ≥ 3) for each experiment, the “All” column refers to the proportion for the cumulative number of neurons across all experiments. (**e**) Mean firing rate (FR) of cells in baseline versus psilocybin condition on a log–log scale for each experiment (exp #1: Wilcoxon signed rank, Z = 4.07, p = 4.77 × 10^–5^**,** exp #2: Wilcoxon signed rank, Z = 2.55, p = 0.0107, exp #3: Wilcoxon signed rank, Z = −3.43, p = 0.0006, exp #4: Wilcoxon signed rank, Z = 2.54, p = 0.0111) and for the total population across all experiments. Overall neurons showed significant activation (Wilcoxon signed-rank, Z = 3*.*75, p = 0.0003, r = 0.32). Data is colour-coded to show individual experiments, with the centre of mass denoted by a cross for each experiment, and the number of neurons in each experimental population shown in the legend. The black cross denotes the centre of mass for total population of neurons across all experiments. All data was analyzed from the 10 min post-psilocybin period described in the “Materials and methods”.

Due to the heterogeneous expression of 5-HT_2A_ and 5-HT_1A_ receptors in neurons of the PFC^12^, the net effect of psilocybin on cortical activity patterns is difficult to infer. We therefore explored the effect of psilocybin administration on spiking activity in the ACC (Fig. 2a). Psilocybin has the highest affinity for 5-HT_2A_ receptors, which, when activated, depolarize neuronal membrane potential. However, psilocybin also activates 5-HT_1A_ receptors which hyperpolarize. The spiking activity of all well-isolated single units (n = 135) was pooled to generate a population firing rate, and baseline and psilocybin conditions were compared (Fig. 2b). Overall, psilocybin introduced more excitation into the network (Fig. 2c), with the mean population firing rate increasing from 34 ± 8 Hz in the baseline condition to 64 ± 19 Hz after drug administration (n = 135). Increased local excitation may be a result of both direct depolarization of pyramidal cells, and release from inhibition via activation of hyperpolarizing 5-HT_1A_ receptors on inhibitory interneurons. Previously, both serotonergic activation^37^, and DOI administration^38,39^, were shown to generate a medley of activation, inhibition and null effects on the ACC population. Here, closer examination of single units after psilocybin administration revealed a similar assortment of responses. Close to half (47%) of the population increased their activity under the psilocybin condition (Fig. 2d), whilst overall, the total neural population across all experiments showed a significant increase in firing rate with a small effect size of r = 0.32 (Fig. 2e) Thus, psilocybin administration is associated with increased activation and a reorganization of neural ensembles in ACC.

### The effect on phase modulation between single unit activity and specific frequency bands

**Figure 3:**
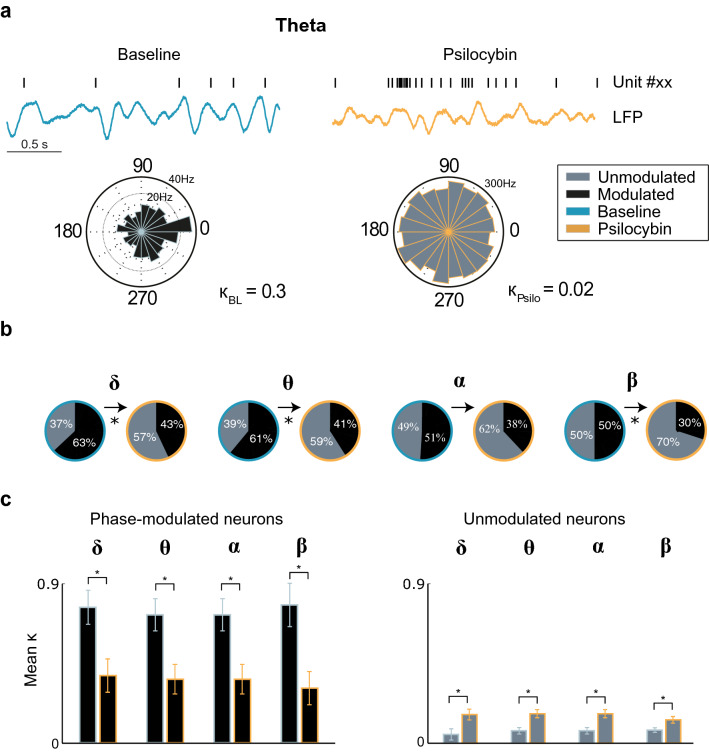
Psilocybin causes loss of phase modulation in low frequency bands. (**a**) Sample trace of band-pass filtered theta band LFP activity, with a concurrent raster plot from a single unit, from baseline and psilocybin conditions. Phase-modulated neuron (κ > 0.2) transitions to an unmodulated state following drug administration. The figure legend applies to all subplots of this figure. (**b**) Pie charts showing the proportion of phase-modulated (black) and unmodulated (grey) cells for delta (chi-square statistic = 8.03, p = 0.0046), theta (chi-square statistic = 7.25, p = 0.0071), alpha (chi-square statistic = 3.42, p = 0.0644), and beta frequency bands (chi-square statistic = 8.33, p = 0.0039), in baseline (blue outline) and psilocybin conditions (orange outline). Those that showed a significant change are denoted with an asterisk. (**c**) Change in mean κ for phase-modulated (left) and unmodulated (right) cells between baseline (blue outline) to psilocybin (orange outline) conditions for delta (phase modulated cells: *t*(68) = 4.39, *p* = 4.01 × 10^–5^, phase unmodulated cells: *t*(51) = 2.68, *p* = 0.0099), theta (phase modulated cells: *t*(66) = 5.52, *p* = 6.23 × 10^–7^, phase unmodulated cells: *t*(53) = −3.17, *p* = 0.0025), alpha (phase modulated cells: *t*(52) = 6.88, *p* = 7.71 × 10^–9^, phase unmodulated cells: *t*(67) = −3.37, *p* = 0.0012, paired sample t-test), and beta frequency bands (phase modulated cells: *t*(51) = 5.38, *p* = 1.91 × 10^–6^, phase unmodulated cells: *t*(68) = −2.87, *p* = 0.0055, paired sample t-test). Data are presented as mean values ± standard error of the mean (SEM).

**Figure 4:**
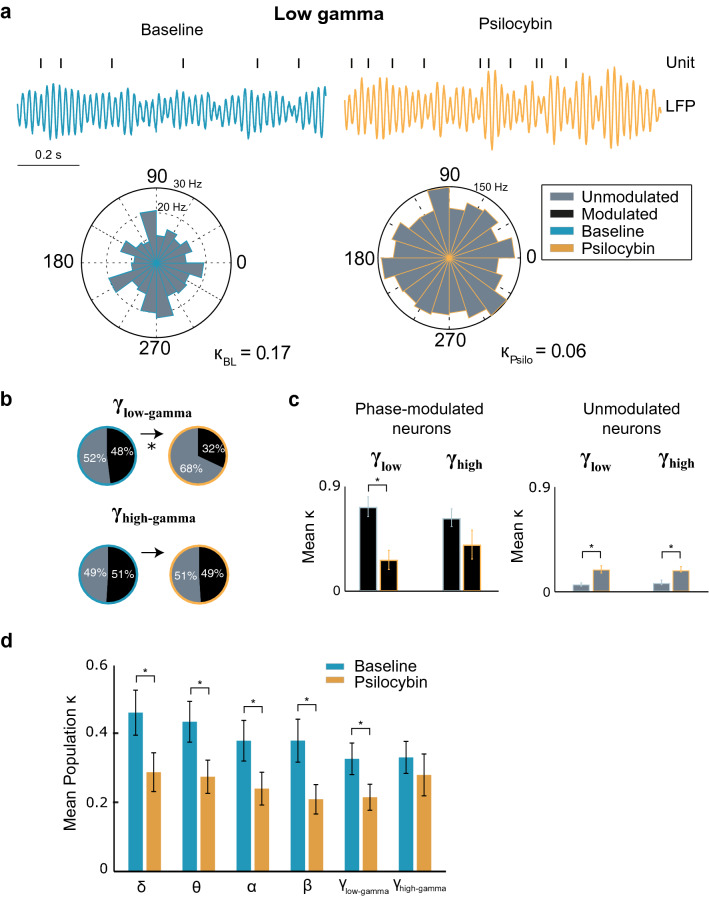
Degradation of phase modulation at higher frequency bands under psilocybin. (**a**) Sample trace of band-pass filtered low-gamma band LFP activity, with a concurrent raster plot from a single unit, from baseline and psilocybin conditions. (**b**) Pie charts showing the proportion of phase-modulated (black) and unmodulated (grey) cells for low gamma (chi-square statistic = 5.33, p = 0.0209) and high gamma (chi-square statistic = 0.08, p = 0.7773) frequency bands in baseline (blue outline) and psilocybin conditions (orange outline). Changes that were significant are denoted with an asterisk. (**c**) Change in mean κ for phase-modulated (left panel) and unmodulated (right panel) cells between baseline (blue outline) to psilocybin (orange outline) conditions for low-gamma (phase modulated cells: *t*(49) = 6.51, *p* = 3.84 × 10^–8^, phase unmodulated cells: *t*(70) = 3.73, *p* = 0.0004, paired sample t-test) and high gamma frequency bands (phase modulated cells: *t*(46) = 1.82, *p* = 0.0749, phase unmodulated cells: *t*(50) = −3.09, *p* = 0.0033, paired sample t-test). (**d**) Mean κ for the total cell population (n = 135) under baseline and psilocybin conditions. All neural frequency bands except high-gamma showed a significant decrease in the degree of phase modulation within the cell population in the post-psilocybin condition (delta: *t*(120) = 2.97, *p* = 0.0036; theta: *t*(120) = 3.59, *p* = 0.0005; alpha: *t*(120) = 3.09, *p* = 0.0025; beta *t*(120) = 3.68, *p* = 0.0116; low-gamma: *t*(120) = 2.56, *p* = 0.0116, high-gamma: *t*(120) = 0.79, *p* = 0.4344, paired sample t-test). Data are presented as mean values ± standard error of the mean (SEM).

Coherence of LFP oscillations between brain areas is thought to facilitate functional interactions between remote areas of the cortex^40,41^. When neurons of a particular cortical region phase lock to a frequency band, it is postulated that this may correspond with an enhanced ability to communicate with neural populations in other cortical areas^29^. There is evidence for a role for phase locking in cognition, as it has been shown in the PFC that phase locking to theta oscillations is enhanced during spatial working memory tasks^29^. Given that psilocybin alters LFP power and neuronal firing rates, we therefore explored whether it also alters phase locking within the ACC. The degree of phase modulation was calculated for each cell (quantified by the concentration of the von Mises distribution, κ), in the baseline and psilocybin conditions for each neural frequency band (Figs. 3a, 4a). The concentration of the von Mises distribution, κ was found to have a significant negative correlation with the p-value of the Rayleigh test for non-uniformity (Pearson correlation coefficient = -0.35, p = 5.27 × 10^–43^). We divided the population into un-modulated (κ < 0.2), and phase-modulated (κ > 0.2) cells. The 0.2 threshold is a conservative heuristic to define phase-modulation^29,30^. Psilocybin produced a significant drop in the fraction of phase-modulated neurons for delta, theta and beta (Fig. 3b) and low-gamma frequency bands (Fig. 4b). This was reflected in a significant decrease in κ for phase-modulated cells for all low frequency bands (Fig. 3c) and the low-gamma frequency band (Fig. 4c). Interestingly, the phase-unmodulated neurons showed a significant increase in κ for both low and high frequency bands (Fig. 3c, 4c). Across the entire population, there was a significant reduction in the mean value of κ for each neural frequency band except high-gamma (Fig. 4d; delta: *t*(120) = 2.97, *p* = 0.0036; theta: *t*(120) = 3.59, *p* = 0.0005; alpha: *t*(120) = 3.09, *p* = 0.0025; beta *t*(120) = 3.68, *p* = 0.0116; low-gamma: *t*(120) = 2.56, *p* = 0.0116, high-gamma: *t*(120) = 0.79, *p* = 0.4344 paired sample t-test), between the baseline and psilocybin conditions. Thus, psilocybin facilitates a transition to a desynchronized state, of which a decrease in power of low frequency bands is also a feature^18^. Psilocybin appears to disrupt phase-locked firing of normally phase-modulated neurons, mediating the transition to a more excitable, but less synchronized network state within ACC.

### Bursting behaviour and phase modulation

**Figure 5:**
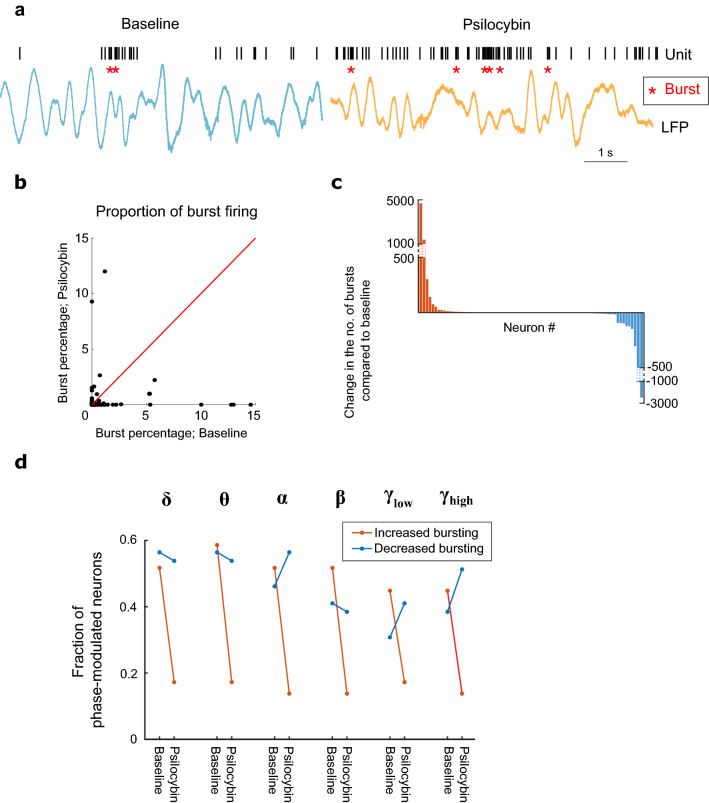
Loss of oscillation-locked bursting activity in ACC under psilocybin. (**a**) Sample trace of band-pass (delta) filtered LFP activity with a concurrent raster plot from a single cell, baseline and psilocybin conditions. Asterisks denote action potential bursts (**b**) The proportion bursting activity per neuron (as a function of total spiking activity) significantly decreased in the psilocybin condition (Wilcoxon signed rank test, Z = 2.51, p = 0.012). (**c**) Number of bursts in the psilocybin condition relative to baseline (n = 135), similar fractions increased and decreased their burst firing activity. (**d**) Significant associations (Fisher’s exact test) were found between baseline phase modulation and the change in bursting activity under psilocybin for all neural frequency bands except low-gamma (Fisher’s exact test, two-tailed: delta p = 0.003, theta p = 0.003, alpha p = 0.000, beta p = 0.031, low-gamma p = 0.062, high-gamma p = 0.002). The proportion of phase modulated cells dropped sharply across neural frequency bands for cells that increased in burst firing following psilocybin.

Within the cortex, high frequency bursting is a more potent means of interneuronal communication than single action potential firing, as it postulated to increase the signal-to-noise ratio of information transfer^42^. Bursts induce synaptic plasticity more efficiently and may selectively represent salient sensory information^43^. We therefore explored whether bursting behaviour was altered in the ACC following psilocybin administration. We defined a burst as two or more consecutive inter-spike intervals of < 6 ms (Fig. 5a). Using this criterion, we found that roughly half (68/135) of ACC neurons exhibited bursting behaviour in either one or both of the baseline and psilocybin conditions. Overall, the proportion of burst firing as a function of total spiking activity per neuron significantly decreased post-drug administration (Fig. 5b). Overall, 30.4% of all neurons increased their proportion of burst firing following psilocybin administration, compared to 20% that decreased (Fig. 5c). Moreover, when we split the population into phase-modulated and unmodulated neurons (based on their baseline κ values), the influence of psilocybin was quite consistent. Whether the neuron increased or decreased in burst firing was significantly associated with whether it was phase-modulated (Fig. 5e). Neurons that increased their number of bursts in the psilocybin condition, were more likely to transition from a phase-modulated to a phase-unmodulated state, as shown by the sharp decrease across all neural frequency bands in the proportion of phase-modulated cells that increased burst firing in the post-psilocybin condition (Fig. 5d). This effect was significant in all neural frequency bands except low-gamma (Fisher’s exact test, two-tailed: delta p = 0.003, theta p = 0.003, alpha p = 0.000, beta p = 0.031, low-gamma p = 0.062, high-gamma p = 0.002). For the associated descriptive matrices for Fisher’s exact test, see Supplementary Fig. S1. Therefore, the incidence of burst firing within the network is redistributed away from phase-modulated cells towards unmodulated cells, and suggests that loss of bursting, perhaps via precise timing of neuronal output, itself may underlie the loss of phase locking that takes place following psilocybin administration.

## Discussion

The examination of the effects of psilocybin on neural networks of the ACC provides a gateway into further understanding how the brain transitions through different states of consciousness. The work presented here investigates the impact of the systemic administration of psilocybin on the ACC of awake head-fixed mice, using multi-unit extracellular recordings. Psilocybin appears to induce a transition of the ACC neural network to a desynchronized state, characterized by a decrease in low frequency oscillation power, with a simultaneous increase in population firing rate, a reduction in the level of phase-modulation with all LFP frequency bands except high-gamma in the network, with a concomitant loss in burst firing that was re-distributed to phase-unmodulated cells. This is consistent with the theory of increased entropy in the brain induced by psilocybin^19^, and the results of the clinical trial on humans whereby psilocybin caused a reduction in power of the alpha frequency band and an increase in power of the gamma frequency band^44^.

Psilocybin reduces the power of low frequency oscillations in the ACC. LFP has a strong role in providing functional connectivity between cortical areas^40,41^. The relative strength of specific neural frequency bands of the LFP can be indicative of information transfer with other brain areas^45^. Whilst gamma oscillations have been proposed to be linked to local networks given that the period of gamma oscillations is closely linked to the decay time of interneuron populations^46^, low frequency oscillations have a sufficiently wide temporal window to facilitate synchrony across distal brain areas. Thus, shifts in patterns of LFP activity can be powerful indicators of changes in information processing/states of the local network, and the reduction in power of low frequency bands may be related to the dissolution of the DMN seen in other studies^15,18^.

Our previous knowledge of the effect of psilocybin on LFP has been reliant on inference from fMRI studies, and intracerebral studies of LFP in rats in response to a similar hallucinogenic compound, DOI. However, the fMRI results are conflicting at present. Studies in rats have shown a decrease in blood flow to the ACC following psilocybin administration^16^. However, earlier PET studies found an increase in cortical metabolism in the ACC^14^. It has been hypothesized that the conflicting results may be due to differing time-courses, with fMRI readings operating on a significantly shorter timescale than PET recordings^15^. fMRI findings in rats also somewhat diverge from that found in humans, however it must be noted that the doses used were different in magnitude. Human data (2 mg/subject) showed a uniform decrease in BOLD activity following psilocybin administration^15^, whereas BOLD signals in rats (2 mg/kg psilocin) increased and decreased depending on cortical area^16^. It must also be noted that the relationship between BOLD signals and LFP has not been definitively established. The findings presented here show a significant decrease in low frequency LFP power and a trend towards an increase in gamma band power in response to psilocybin (Fig. 1d). This is in agreement with the fMRI findings stated above, the results of DOI investigations in the ACC^39^, and the findings of psilocybin administration in humans^24^.

### Psilocybin increases the activation level of cell populations in the ACC

The effect of endogenous serotonin on the PFC can be seen in experiments examining the effects of activating the raphe nuclei^37^. Stimulation of the raphe nuclei caused a medley of effects on the ACC, with 66% of pyramidal cells being inhibited, 13% being excited, and 20% showing a bi-phasic response. This may be dependent on the relative activation of 5-HT_2A_ and 5-HT_1A_ receptors, as it has been shown that these receptors induce depolarization and hyperpolarization respectively^9,10^, and that a portion of PFC neurons show co-expression of the two receptors^11^. The results presented here echo this disruption in the balance of excitation and inhibition in the network. Over 2/5 of cells were significantly activated in response to psilocybin administration (Fig. 2d).

### Decrease in network synchrony in response to psilocybin administration

The increase in cell population firing rates with a simultaneous decrease in power of low frequency oscillations is accompanied by an overall reduction in phase modulation with each neural frequency band except high-gamma (Fig. 4d). This echoes the previous findings in rats whereby a 1 mg/kg dose DOI was found to de-synchronize neuronal firing activity with gamma oscillations^39^ and the active phase of the LFP^38^. Whilst previously un-modulated cells show a significant, yet modest increase in their phase modulation strength, the average phase modulation strength of modulated cells decreases strongly for all frequency bands except high gamma (Figs. 3c, 4c). Moreover, an association was found between the change in burst firing induced by psilocybin and whether the cell is phase-modulated or unmodulated. The reduction in power of low frequency bands may be symptomatic of the dissolution of the default mode network^15,18^. The desynchronization of the local ACC network, and the relationship with the change in bursting activity, appear to indicate that psilocybin increases entropy in the network. This could lead to interesting areas of future research into how the PFC governs top-down processing and how psilocybin may disrupt this activity.

## Conclusions

Administration of psilocybin disrupts excitation/inhibition balance in the ACC and is accompanied by desynchronizaction of single unit activity with respect to LFP oscillations. This may reflect the decrease in functional connectivity between brain areas observed in fMRI studies of psilocybin administration in humans^15^. It is worth noting that these results are in agreement with that of DOI studies that found that DOI decreased phase modulation of neurons with gamma oscillations and the active phase of the LFP^38,39^. Furthermore, the incorporation of the effects on the relative power in the LFP would suggest that psilocybin induces a transition to a desynchronized cortical state in the ACC, as previously postulated^18,19^. A desynchronized state is characterized by a decrease in low frequency power and an increase in gamma oscillatory power^47^. The systemic administration of psilocybin caused a similar decrease in power of low frequency oscillations and a trending increase in gamma oscillatory power. These findings would indicate that psilocybin is inducing a state of desychronized cortical activity that may be indicative of the disruption of top-down processing that is postulated to be the mechanism of action of psychedelic compounds, as put forward by the Relaxed Beliefs Under Psychedelics (REBUS) model^48^.

## Supplementary Information

Supplementary Figure S1.

## Data Availability

Data and Matlab code associated with this study are available on request from the corresponding author.
