# Supplementary material for: Psilocybin reduces low frequency oscillatory power and neuronal phase-locking in the anterior cingulate cortex of awake rodents
Source: Sci Rep. 2022 Jul 26;12:12702. doi: 10.1038/s41598-022-16325-w (PMC9325720; doi:10.1038/s41598-022-16325-w)
Supplement: Supplementary file 1 — Supplementary Figure S1. [file 41598_2022_16325_MOESM1_ESM.pdf]

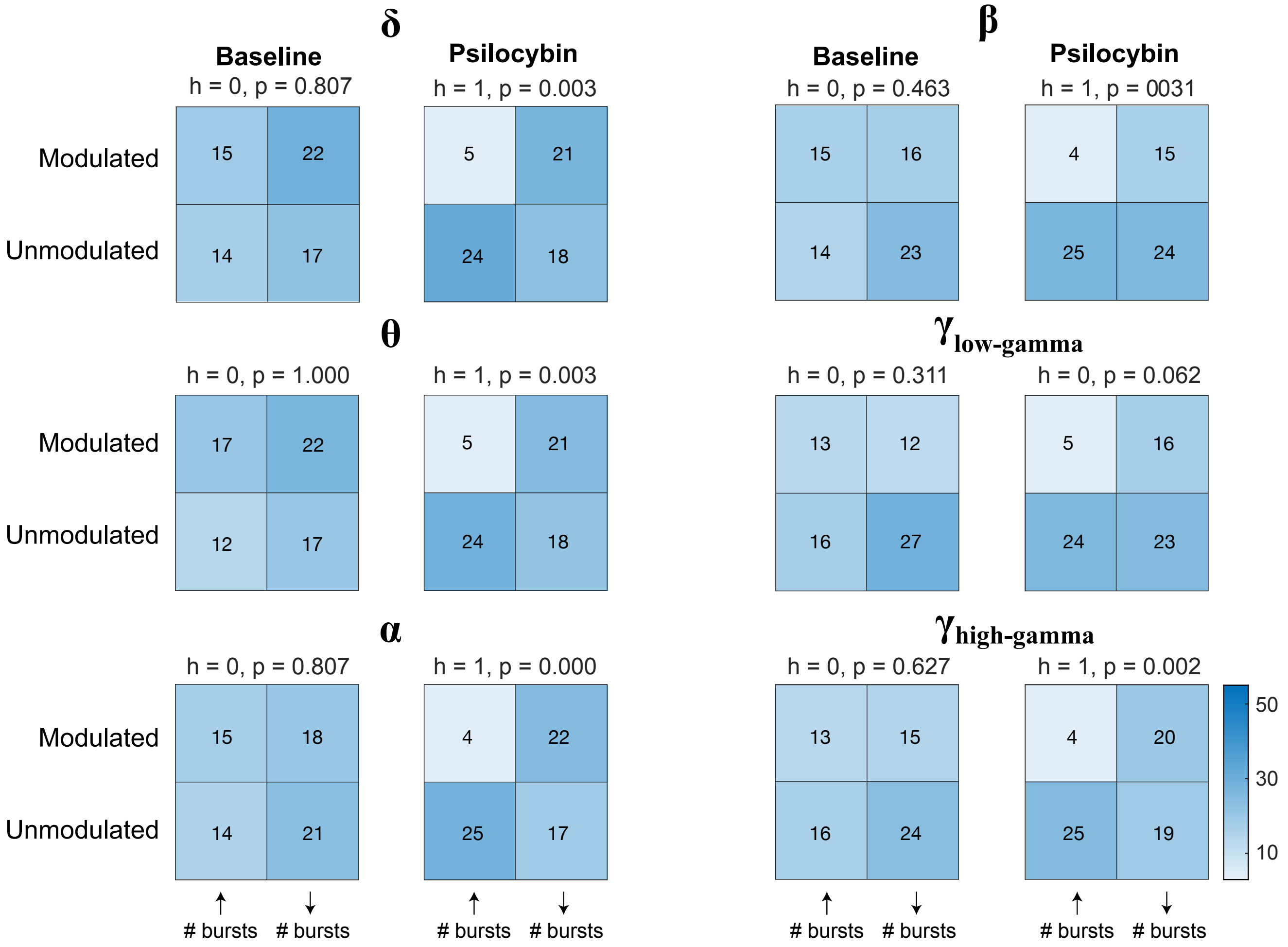

Supplementary Figure S1: The descriptive statistics are shown for the distribution of phase modulated and unmodulated cells that increased and decreased bursting respectively following psilocybin administration. The baseline period values are shown in the tables on the left-hand-side, and the post-psilocybin period values are shown in the tables on the right-hand-side. Fisher's exact test was used to examine if there was an association between whether cells were phase modulated or unmodulated and a change in burst firing (two-tailed: delta p = 0.003, theta p = 0.003, alpha p = 0.000, beta p = 0.031, low-gamma p = 0.062, high-gamma p = 0.002).
